# Utility of using far‐field R‐wave signals in the detection of fatal ventricular arrhythmia

**DOI:** 10.1002/joa3.13207

**Published:** 2024-12-23

**Authors:** Yousaku Okubo, Hisayasu Matsuzaki, Shogo Miyamoto, Sho Okamura, Yukiko Nakano

**Affiliations:** ^1^ Department of Cardiovascular Medicine Hiroshima University Graduate School of Biomedical and Health Sciences Hiroshima Japan; ^2^ Division of Clinical Engineering, Clinical Support Department Hiroshima University Hospital Hiroshima Japan

**Keywords:** implantable cardioverter‐defibrillators, sudden cardiac death, ventricular fibrillation, ventricular fibrillation therapy assurance, ventricular tachycardia

## Abstract

Current guidelines recommend cardioverter‐defibrillator (ICD) programming, including faster detection rates, longer detection durations, and strict discrimination for supraventricular tachycardia (SVT) to prevent unnecessary ICD treatment. This delayed‐style ICD programming could lead to a rise in the possibility of VF undersensing. To avoid this risk, an innovative algorithm known as VF Therapy Assurance (VFTA; Abbott, Sylmar, CA) has been developed. VFTA uses far‐field R‐wave signals during VT or VF episodes to provide ICD therapy in cases of near‐field R‐wave signal undersensing.

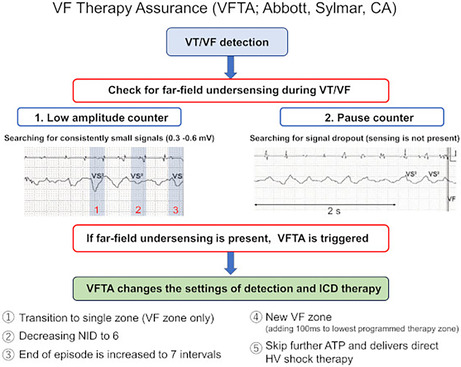

Implantable cardioverter‐defibrillators (ICDs) are well‐established for the primary and secondary prevention of sudden cardiac death (SCD) because of fatal ventricular arrhythmias.

However, although rarely, SCD can occur after ICD implantation. Furthermore, Tseng et al. revealed that undersensing ventricular tachycardia (VT) or ventricular fibrillation (VF) or failure to detect the same was found in five out of seven patients with ICDs who had SCD because of VT or VF.^1^ Chaotic irregular RR intervals and R‐wave amplitude variations during VF may cause VF undersensing. Moreover, current guidelines recommend ICD programming, including faster detection rates, longer detection durations, and strict discrimination for supraventricular tachycardia (SVT) to prevent unnecessary ICD treatment.^2^ This delayed‐style ICD programming could lead to a rise in the possibility of VF episode undersensing. To avoid this risk, an innovative algorithm known as VF Therapy Assurance (VFTA; Abbott, Sylmar, CA) has been developed. VFTA uses far‐field R‐wave signals during VT or VF episodes to provide ICD therapy in cases of near‐field R‐wave signal undersensing. Herein, we present a rare case in which the VFTA algorithm leads to the early detection and successful treatment of fatal ventricular tachycardias for which ICD therapy would otherwise be delayed or deferred.

An 82‐year‐old man with a reduced left ventricular ejection fraction of 28% because of old anterior wall myocardial infarction and persistent atrial fibrillation underwent single‐chamber ICD (Gallant™; Abbott, Sylmar, CA) implantation for secondary prevention of VF. RV shock lead was placed in the RV septum away from the scar tissue area because of the myocardial infarction. Furthermore, all the tested lead parameters were normal with an R‐wave amplitude of at least 12 mV during intrinsic rhythm. He was remotely monitored on Merlin.net™ (Abbott, Sylmar, CA). ICD therapy programming was set at two tachycardia rate zones: (1) the VT1 zone (>150 bpm/400 ms; number of intervals to detect [NID], 16 intervals) and (2) the VF zone (>230 bpm/260 ms; NID, 12 intervals). Six months after ICD implantation, he visited our hospital because of a remote VF event alert. In this VF episode, the VFTA was activated, and early VF detection, which could have delayed detection, resulted in appropriate high‐voltage (HV) shock therapy.

Initially, the tachycardia episode above the VT1 rate (>150 bpm) started with regular tachycardia (Figure 1A). Of the SVT discrimination algorithm, the sudden onset criterion identified tachycardia as VT, while the other rate stability and morphology criteria identified it as SVT, resulting the diagnosis of SVT. However, while the tachycardia was sustained, the morphology changed and the rate stability increased; thus, the tachycardia was diagnosed as VT. Subsequently, antitachycardia pacing (ATP) was performed (Figure 1B). Although the tachycardia changed from VT to VF after a second ATP therapy was performed, the device failed to diagnose VF, and a third and fourth ATP was performed (Figure 1C). Despite optimal RV sensing, intermittent undersensing during VF occurred. Then, HV shock therapy for VF was triggered at the sixth VF annotation, even though the NID did not meet the VF criteria (NID = 12). Nine seconds after VF detection, the device delivered a 36‐joule shock that effectively converted the VF rhythm and restored an intrinsic rhythm (Figure 1C,D). The reason why VF therapy was triggered after only six beats was VFTA activation. The area circled in red in Figure 1C shows that over 2 s, there was no potential recorded in the discrimination channel that monitored the far‐field R‐wave signals, suggesting that far‐field R‐wave undersensing had occurred. The VFTA employs two undersensing criteria that are continuously monitored on the secondary far‐field sensing channel, and it is triggered when either one meets the following criteria: (1) consecutive, low‐amplitude R waves (<0.6 mV) and (2) excessively long R–R intervals (>2 s). The individual counters are increased when low‐amplitude R waves or long R‐R intervals are detected. Large signals (>1 mV) can also reset the counter to zero. The VFTA triggers if the low‐amplitude counter is ≥2 or the pause counter is ≥1. The VFTA changes the settings of detection and ICD therapy as follows: (1) transition to a single zone (the VF zone only) with a slower rate cut‐off (adding 100 ms to the slowest programmed therapy zone up to a maximum of 400 ms); (2) decreasing the NID to 6; (3) increasing the number of binned sinus intervals required to end of the episode from five to seven; and (4) skips further ATP and delivers direct HV shock therapy. The detailed mechanism and settings of the VFTA have previously been reported.^3^ Table 1 shows the device binning after VFTA activation. Per the binning rule, the current interval, and average interval (which is the average of the current interval and the three previous R–R intervals) were used to make the determination. The 5th, 10th, and 12th beats from the top (marked with an asterisk) are noteworthy. If the VFTA was not activated (in the primary normal settings), these episodes would not have been judged as VF. The nonactivation of the VFTA could have resulted in delayed or disabled VF detection. The VFTA was also triggered 2 months after this episode (Figure 2). Here, the VFTA was triggered by the low‐amplitude counter meeting the criteria, and HV therapy was provided early.

**FIGURE 1:**
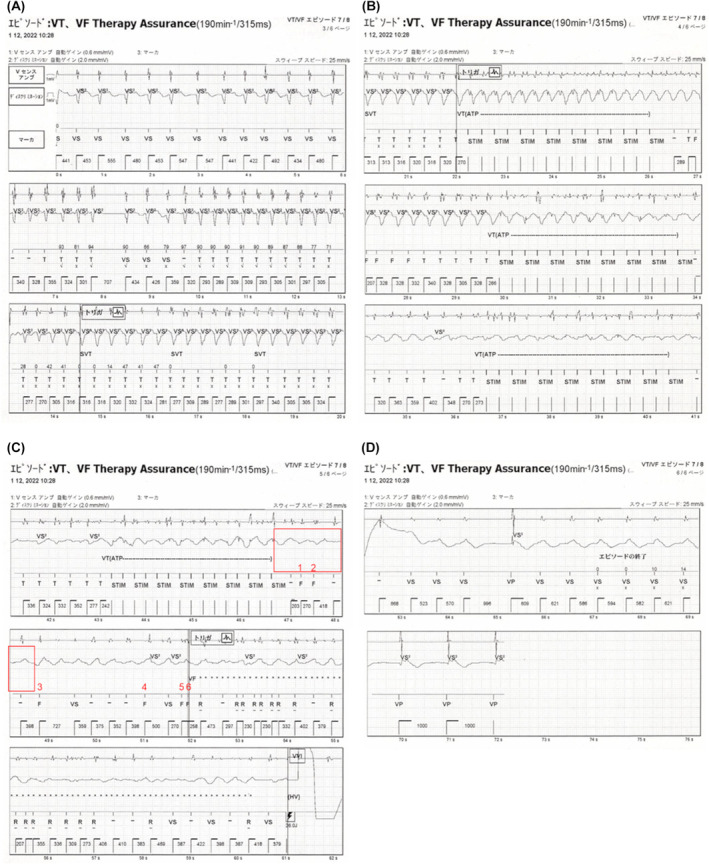
(A) Tachycardia episode onset. (B) Initial ventricular tachycardia detection by the device. (C) VF Therapy Assurance (VFTA; Abbott, Sylmar, CA) activation and high‐voltage shock therapy at 36 joules. (D) End of the episode (return to an intrinsic rhythm) after high‐voltage shock therapy. ATP, antitachycardia pacing; F, ventricular interval within ventricular fibrillation zone; HV, high‐voltage shock therapy; R, reconfirmed interval; STIM, stimulus of antitachycardia pacing; SVT, supraventricular tachycardia; T, ventricular interval within ventricular tachycardia zone; VF, ventricular fibrillation; VS, sinus ventricular interval; VS2, low‐amplitude R‐wave signal detected by the coil‐to‐can sensing channel (discrimination channel); VP, ventricular pacing; VT, ventricular tachycardia.

**TABLE 1:** Device binning after VF Therapy Assurance (VFTA; Abbott, Sylmar, CA) activation.

Current interval (CI)	Average interval (AI)	Binned
203	269	(VF‐VT) = VF
270	248	(VT‐VF) = VF
418	283	(NSR‐VT) = discarded
420	328	(NSR‐VT) = discarded
398	377	(VF‐VF) = VF*
727	491	(NSR‐NSR) = VS
359	476	(VF‐NSR) = discarded
375	465	(VF‐NSR) = discarded
352	453	(VF‐NSR) = discarded
398	371	(VF‐VF) = VF*
500	406	(NSR‐NSR) = VS
270	380	(VF‐VF) = VF*
150	330	(VF‐VF) = VF

*Note*: The binning method classifies ventricular arrhythmias detection by current and average intervals.

Abbreviations: NSR, normal sinus rhythm; VF, ventricular fibrillation; VS, sinus ventricular interval; VT, ventricular tachycardia.

**FIGURE 2:**
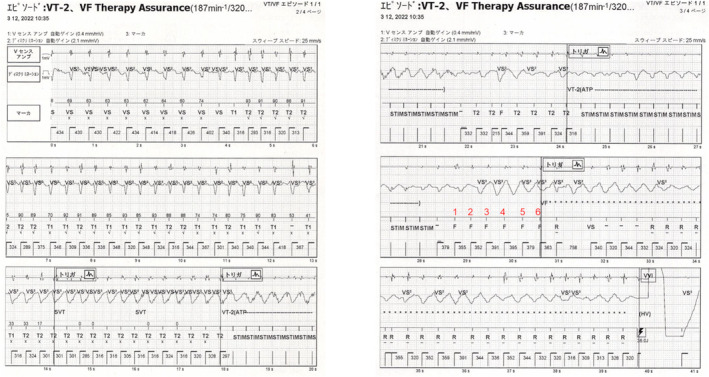
Onset of ventricular tachycardia, VF Therapy Assurance (VFTA; Abbott, Sylmar, CA), and appropriate high‐voltage shock therapy at 36 J. Abbreviations as in used in Figure 1.

Currently, defibrillation threshold testing (DFT) is no longer routinely recommended for transvenous ICD implantation, making it difficult to predict VF undersensing. A previous study showed possible occurrence of some undersensing of the VF episode, even with large R‐wave amplitudes during sinus rhythm at the implant.^4^ This is because of the inherent nature of polymorphic VT (PVT) and VF considering the dynamically changing ventricular signal amplitude and intervals. However, Lillo‐Castellano et al. showed that baseline R‐wave amplitudes ≤2.5 mV in patients with ICDs may lead to a high risk of delayed VF detection.^5^ Therefore, conditions that result in low R‐wave amplitudes at the implant or during follow‐up (e.g., arrhythmogenic right ventricular dysplasia, cardiac sarcoidosis, and cardiac amyloidosis) may benefit from the VFTA.

Recent aggressive delayed‐style ICD programming has increasingly raised the possibility of VF underdetection, and the VFTA is a promising algorithm that can mitigate this risk by enabling the prompt detection and treatment of potentially fatal ventricular arrhythmias.

## CONFLICT OF INTEREST STATEMENT

Authors declare no conflict of interests for this article.

## ETHICS APPROVAL STATEMENT

Approval was obtained from the local ethics committee.

## INFORMED CONSENT

The authors obtained informed consent from the patient.

## PATIENT CONSENT STATEMENT

The authors obtained consent from the patients.

## PERMISSION TO REPRODUCE MATERIAL FROM OTHER SOURCES

Available upon reasonable request.

## Data Availability

Available upon reasonable request.
